# Curative Intent Radiation for Anal Cancer in Pelvic Kidney Transplant: A Case Report With an Eight-Year Follow-Up

**DOI:** 10.7759/cureus.46366

**Published:** 2023-10-02

**Authors:** Alexandre F Riopel, Lucy Ward, Nikhilesh Patil

**Affiliations:** 1 Department of Radiation Oncology, Dalhousie University at Queen Elizabeth II Health Sciences Centre, Halifax, CAN; 2 Department of Medical Physics, Dalhousie University at Queen Elizabeth II Health Sciences Centre, Halifax, CAN

**Keywords:** radical radiotherapy, three-dimensional conformal radiotherapy, pelvic external beam radiotherapy, vmat radiotherapy, transplant kidney, pelvic radiotherapy, anal canal cancer, radiation and clinical oncology

## Abstract

The incidence of malignancies seen after solid organ transplant is increasing, and oncologists are seeing more patients with transplanted organs. In this case report, we present how pelvic radiotherapy can be safely administered in a patient with a transplanted kidney by conducting a comprehensive chart review and analyzing the dosimetry in the radiotherapy planning software Eclipse. A 52-year-old female patient received a kidney transplant in 2002 and was diagnosed 11 years later with a cT3 N0 M0 squamous cell carcinoma of the anal canal. She was offered radical radiation therapy with 45 Gy in 25 fractions using a volumetric modulated arc therapy plan to the pelvic lymph nodes and tumor followed by a 9-Gy boost to the anal tumor alone using a three-dimensional conformal radiation therapy plan with concurrent 5-fluorouracil/mitomycin chemotherapy for a total dose of 54 Gy. The right external iliac and inguinal lymph nodes coverage was compromised to decrease the solitary pelvic kidney dose in addition to creating a 1-cm planning risk volume around the kidney and using half-beam blocks. Her pelvic kidney only received a mean dose of 6.68 Gy. Eight years later, the patient continues to be cancer-free, as evident with a recent sigmoidoscopy in 2021 and a physical examination in 2022. Her creatinine started to rise one year post-treatment, but age of the transplanted kidney is likely the cause of kidney failure.

## Introduction

The overall risk of developing cancer following a solid organ transplant is increased twofold and can represent an excess absolute risk of 0.7% per year for a broad spectrum of infection-related or non-infection-related malignancies [1]. Inherently, oncologists will see more patients with transplanted organs as more transplantations are being performed, immunosuppressive therapy is improving, and patients are living longer [1-4].

There is an increase in the relative risk of anal cancer or anal dysplasia in the kidney transplant population [2,5]. Radiation techniques have dramatically improved over the past decade and remain a treatment of choice for anal cancer [6]. A pelvic kidney can be a challenge in the treatment delivery especially when treating the pelvic lymph nodes. A mean dose of less than 4 Gy to the transplanted kidney is suggested to decrease the chance of organ failure [7]. To the best of our knowledge, this is the first case report describing the treatment of an anal carcinoma using concurrent chemoradiation in a pelvic transplanted kidney.

The patient provided written informed consent for this case report.

## Case presentation

Clinical details

The patient was a 52-year-old female with a history of polycystic kidney disease, which was also affecting her liver, resulting in occasional pleural effusion and ascites. She received a living-related kidney transplant in 2002 for kidney failure and required a left hepatectomy, cholecystectomy, and bilateral nephrectomy due to mass effect in 2009. She sought medical attention in late 2013 for rectal bleeding and rectal pain, worsening over several weeks, in addition to a narrowing of her stools.

At the time of our assessment, she was on FK-506, hydrochlorothiazide, amlodipine, vitamin D, and calcium and reported being otherwise healthy with an ECOG (Eastern Cooperative Oncology Group) performance status grade 1. Physical examination revealed no palpable cervical, supraclavicular, axillar, or inguinal lymphadenopathy. The abdomen was soft but with significant hepatomegaly. The gynecological examination was normal for the cervix or vagina. The anal examination showed a posterior ulcerated 5x5x6 cm tumor with good rectal tone. The anterior rectal wall was spared without perianal involvement.

She subsequently underwent a biopsy that demonstrated an invasive well-differentiated squamous cell carcinoma of the anal canal. A non-enhanced computed tomography (CT) of the chest, abdomen, and pelvis, and a positron emission tomography (PET) scan did not show evidence of lymphadenopathy or metastatic disease and confirmed a 5.8-cm mass in the craniocaudal dimension in the anal canal, extending from the anal verge. Her baseline blood workup prior to starting radiation was as follows: hemoglobin of 109 g/L, white blood count of 5.9x10^9^/L, platelet of 215x10^9^/L, creatinine of 127 umol/L, urea of 6.3 mmol/L, tacrolimus level of 5.2 ng/mL, and electrolytes within normal limits.

Her final staging was cT3 N0 M0 as per the 7th edition of the AJCC (American Joint Committee on Cancer) staging system, which was in use in 2014 [8,9]. She was offered 54 Gy of radical external beam radiotherapy with concurrent 5-fluorouracil/mitomycin chemoradiation, which is the standard of care for anal cancer at our center.

Radiation planning and treatment delivery

A planning CT of the abdomen and pelvis without contrast was obtained with a 2.5-mm slice thickness. The patient was in the supine position using a knee/foot fix system and a Vac Lok from the mid-abdomen to the mid-thigh. No bolus was required as there was no perianal involvement. The patient was instructed to empty her bladder and to drink 500 mL of water 45 minutes before the planning CT and subsequent treatments. No treatment was delivered if the gas in the rectum was more than 5 cm in diameter. Eclipse v10 (Varian, Palo Alto, CA) was the treatment planning system used. The planning CT was fused with the diagnostic CT and PET scans to assist with delineation of the target. The organs at risk that were contoured included the pelvic kidney, bladder, rectum, liver, lower spinal cord, bilateral femoral heads, bone marrow, bowel bag, and external genitalia. The anal mass was contoured as gross tumor volume (GTV). A clinical target volume (CTV) was then created using the GTV with a 1-cm radial and 1.5-cm cranio-caudal expansion. A CTV was created for the elective nodes including the mesorectal, inguinal, internal iliac, and external iliac bilaterally as well as a modified nodal contour, compromising on the right external iliac and inguinal coverage. A planning target volume (PTV) was created using an 8-mm expansion around the CTV. A PRV consisting of a 1-cm expansion of the pelvic kidney was created to assist in decreasing the dose to this organ during the planning phase.

Three planning attempts were made comparing the original CTV nodal coverage to the modified nodal coverage. The initial plan was a volumetric modulated arc therapy (VMAT) simultaneous integrated boost (SIB) technique with 45 Gy in 25 fractions to the elective nodal volume delivered simultaneously with a total dose of 54 Gy in 25 fractions to the primary disease site. The best achievable maximal kidney dose was 36.9 Gy with a mean dose of 11.6 Gy. The second attempt used a VMAT SIB technique lowering the dose to the PTV nodes to 40 Gy. The best achievable maximal kidney dose was 16.1 Gy with a mean dose of 6.89 Gy without using avoidance arcs as it was not commissioned at our center in 2014. Therefore, a third and final attempt was made to spare the kidney maximally.

The final and approved plan used a single-level VMAT delivering 45 Gy in 25 fractions in the first phase to the pelvic lymph nodes and tumor. The second phase delivered 9 Gy in five fractions to the anal primary tumor alone using a three-dimensional conformal radiation therapy (3D-CRT) plan with a half-beam block technique using six fields. In total, 54 Gy in 30 fractions was delivered to the primary disease site.

**Figure 1:**
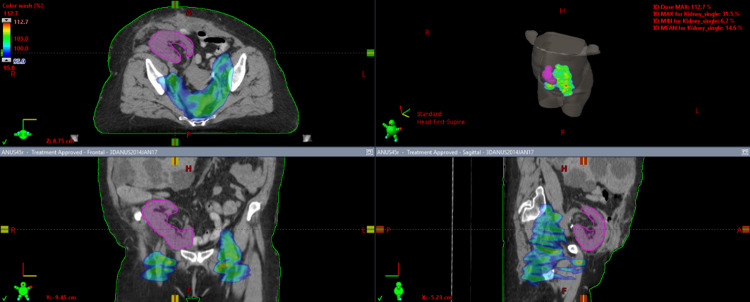
Qualitative dose distribution of phase 1 VMAT plan at 95% of the prescription dose of 45 Gy; the area encompassed with the blue-green shade marks 42.75 Gy. VMAT, volumetric modulated arc therapy

**Figure 2:**
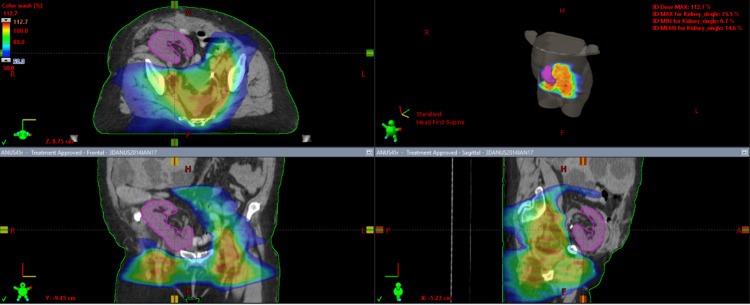
Qualitative dose distribution of phase 1 VMAT plan at 50% of the prescription dose of 45 Gy; the area encompassed with the blue-green shade marks 22.5 Gy. VMAT, volumetric modulated arc therapy

**Figure 3:**
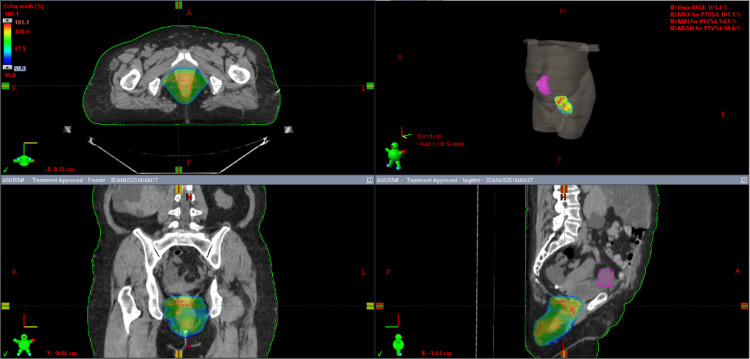
Qualitative dose distribution of the phase 2 3D-CRT plan at 95% of the prescription dose of 9 Gy; the area encompassed with the blue-green shade marks 8.55 Gy. 3D-CRT, three-dimensional conformal radiation therapy

**Figure 4:**
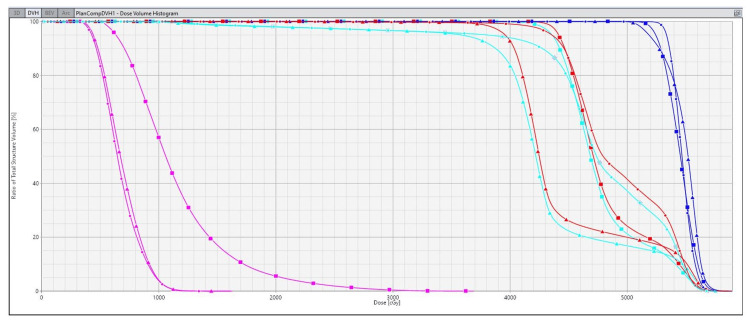
Dose volume histogram of the three plans with the original and the modified nodal contours. The pelvic kidney dose is shown in pink, the original nodal PTV node is shown in turquoise, the modified nodal PTV is shown in red, and the PTV 54 Gy is shown in blue. The square represents the first plan delivering 45 Gy in 25 fractions to the elective nodal volume with a total dose of 54 Gy in 25 fractions to the primary disease site using VMAT and SIB. The triangle represents the second attempt using VMAT SIB technique lowering the dose to 40 Gy to the PTV nodes, with a total dose of 54 Gy to the primary disease site. The circle represents the third attempt using a single-level VMAT delivering 45 Gy in 25 fractions in the first phase to the pelvic lymph nodes and tumor with a second phase of 9 Gy in five fractions to the anal primary tumor alone using a 3D-CRT plan with a half-beam block technique. 3D-CRT, three-dimensional conformal radiation therapy; PTV, planning target volume; SIB, simultaneous integrated boost; VMAT, volumetric modulated arc therapy

A composite plan of the VMAT and 3D-CRT plans was generated showing the final achievable maximal kidney dose of 16.3 Gy with a mean kidney dose of 6.68 Gy using the modified nodal contour. The composite plan target volumes received a minimum of 95% dose coverage for both the modified PTV 45 Gy and the PTV 54 Gy. The maximum volume within the PTV equivalent to 0.03 cc did not receive more than 107% of the prescribed dose. The dose wash of the third plan as well as the dose volume histogram (DVH) of the three plans can be visualized in Figures 1-4.

A cone beam CT of the pelvic bone auto match was first used followed by a PTV and kidney soft tissue match for phase 1 plan with VMAT. A bony match with kv imaging was used for the phase 2 3D-CRT plan. The couch shift mean in absolute value for the vertical, longitudinal, and lateral axis was respectively 20 mm, 23 mm, 21 mm for phase 1 and 30 mm, 26 mm, and 28 mm for phase 2.

The patient tolerated the treatment well overall but required a two-week break between phase 1 and 2 secondary to skin and mucous membrane toxicity and significant fatigue. She also reported a decreased oral intake, mild constipation, mild pain, mild urinary frequency/urgency, and one episode of nausea. She completed treatment at the end of March 2014 after eight weeks including the two-week break.

Follow-ups and creatinine levels

After external beam radiotherapy, there was no change in her creatinine levels, and a PET scan performed six months after her radiation was negative for malignancy. She was last seen in our clinic close to the five-year mark following her oncology treatment. She continued to be followed in the community and had a negative biopsy for malignancy while undergoing investigation for hemorrhoids. She underwent a sigmoidoscopy and a CT colonoscopy in 2021 without evidence of recurrence while investigating for rectal bleeding secondary to being on anticoagulant. An experienced surgeon performed a physical examination in 2022, which revealed post-radiation changes without evidence of recurrence.

**Figure 5:**
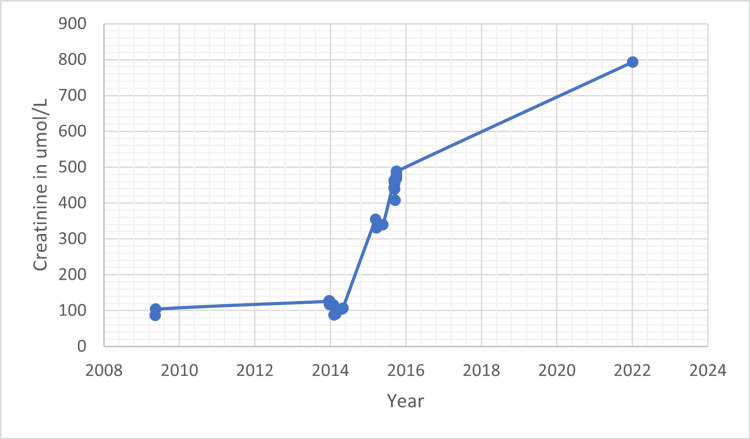
Creatinine level (in umol/L).

As seen in Figure 5, her creatinine was around the 100’s umol/L in 2009 and started rising shortly prior and one year after the radiation treatment. In 2022, 22 years post-transplant, the patient is in kidney failure with a creatinine level in the 700’s umol/L and is receiving dialysis. Her creatinine is not available prior to 2008, between 2009 and 2013, and between 2015 and 2022.

## Discussion

The decision was made to treat with the third plan combining VMAT, 3D-CRT, and a modified elective nodal volume as it gave the lowest mean kidney dose while achieving the appropriate coverage for the PTV volumes. The plan objective to cover the target volumes by 95% of the prescribed doses was achieved with this plan using 45 Gy for the nodal volume, which was the standard of care at our institution [10,11]. Delivering 40 Gy to the nodal volume with the second attempt could have compromised her outcomes as salvage treatment in the pelvis following radiotherapy is challenging.

Compromising coverage on the nodal volume was deemed appropriate as the patient had N0 disease, and renal tolerance depends on the irradiated volume of the organ [12]. The risk of radiation nephropathy would be a critical complication in a patient with a transplanted pelvic kidney despite the high requirements of curative intent radiation plans. Structural changes such as mesangiolysis, sclerosis, tubular atrophy, and tubulointerstitial scarring in addition to hypertension and anemia can be seen with radiation nephropathy, which can lead to a loss of function [13].

To avoid this complication, QUANTEC suggests that the maximal mean dose that a native kidney can get for a less than 5% risk of kidney injury for bilateral native kidneys is 18 Gy; alternatively, if one kidney receives more than a mean dose of 18 Gy, the contralateral native kidney should not receive more than 6 Gy to more than 30% of its volume [14]. However, Dahlke et al. described that the tolerance dose for a transplanted kidney exposed to immunosuppression may be lower and it should be limited to a mean dose of less than 4 Gy [7]. In this case, this was not possible to achieve without further compromising the nodal volume and potentially the cure rate. A mean dose of 6.68 Gy as achieved in the third planning attempt was felt appropriate as per our institutional dose constraints guidelines and was therefore approved for treatment.

Using VMAT and 3D-CRT enabled the dosimetrist to protect the kidney. VMAT plans have a larger low dose wash, which is why 3D-CRT technique with a half-beam block can be advantageous when trying to boost a critical structure. Additionally, having only one field with the kidney on exit dose assisted in limiting the kidney dose further. A PRV of 1 cm around the kidney assisted in keeping the dose away from the kidney in the planning process and was crucial to the successful delivery of this radiation treatment.

Eight years post-treatment, the patient continues to do well without any evidence of recurrence after physical examinations, biopsies, PET scans, and CT scans.

Unfortunately, her kidney started to fail with a creatinine in the 700’s umol/L after 22 years of transplant. She is currently receiving dialysis. In the late 1990s and early 2000s, the overall one-year and projected 10-year graft survival rates for living donor transplants were 95% and 68%, respectively [15]. Living kidney donor transplant median survival was 12.1 years between 1995 and 1999 [16]. She received her kidney transplant in 2002, and therefore the cause of her kidney failure is likely to be the age of the kidney as she started her radiation treatment in 2014, 12 years post-transplant. Her creatinine started increasing slightly prior to the treatment and more significantly in 2015.

An active malignancy is an absolute contraindication to receiving a kidney transplant, whereas a history of previous radiation or a successfully treated malignancy is not [17]. Therefore, a second kidney transplantation could be considered, emphasizing the importance of careful planning of curative intent treatment in a patient with a pelvic transplanted kidney. Maximizing oncological outcomes and protecting the kidney should be weighted according to the staging and salvage options.

## Conclusions

With the advancement of modern medicine, patients with transplanted organs live longer. With an increased risk of developing cancer while immunocompromised, radiation oncologists will face challenges when delivering radiation treatments to preserve the transplanted organ’s function. This case report outlines the successful delivery of a curative intent treatment to a patient with T3 N0 M0 anal cancer and in the presence of a transplanted pelvic kidney. When carefully planned, combining VMAT and 3D-CRT planning techniques in addition to modifying the elective nodal contour and creating a 1-cm PRV to minimize the dose to the pelvic kidney was an effective way to reduce kidney exposure. Age of the pelvic kidney is likely the cause of kidney failure for this patient, and she remains cured after eight years of follow-up with physical examinations, direct visualization, and imaging.
